# Supplementary material for: Adipocyte metabolism is improved by TNF receptor-targeting small RNAs identified from dried nuts
Source: Commun Biol. 2019 Aug 21;2:317. doi: 10.1038/s42003-019-0563-7 (PMC6704100; doi:10.1038/s42003-019-0563-7)
Supplement: Supplementary file 1 — Supplementary Information [file 42003_2019_563_MOESM1_ESM.docx]

**Supplementary Information**

**
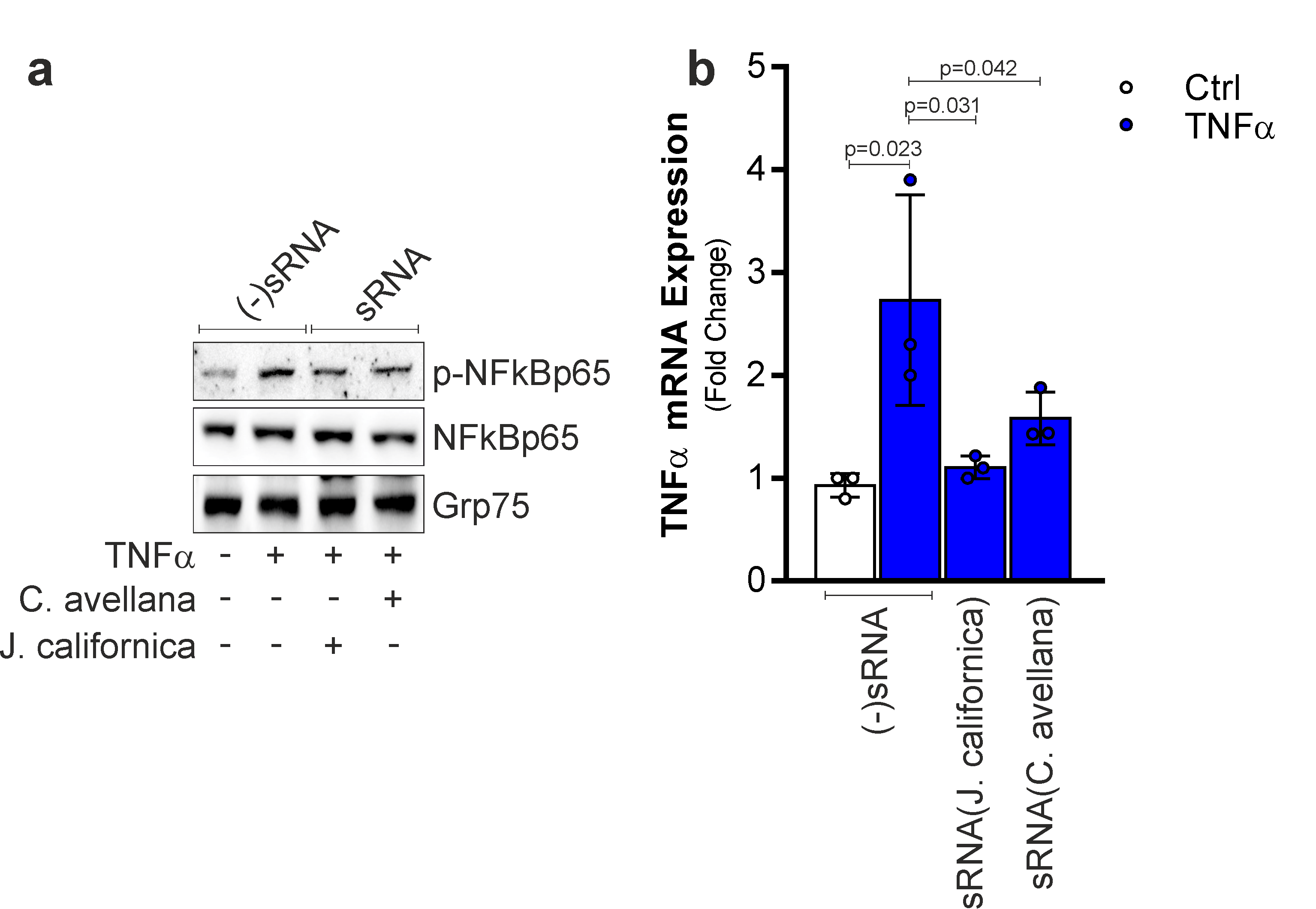
**

**Supplementary Fig. 1. Small RNA extracted from nuts limit inflammatory pathway in adipocytes.**

(**a, b**) p-NFkBp65 protein (a) and TNF-α mRNA (b) levels were analyzed in TNF-α-treated adipocytes and transfected with a pool of small RNA (sRNA) isolated from *J. californica* and *C. avellana*. Transfection with a scramble small RNA [(-)sRNA] was used as negative control. All immunoblots reported are representative of three independent experiments. Grp75 and NFkBp65 were used as loading controls. Data are expressed as means ± S.D. (n = 3).


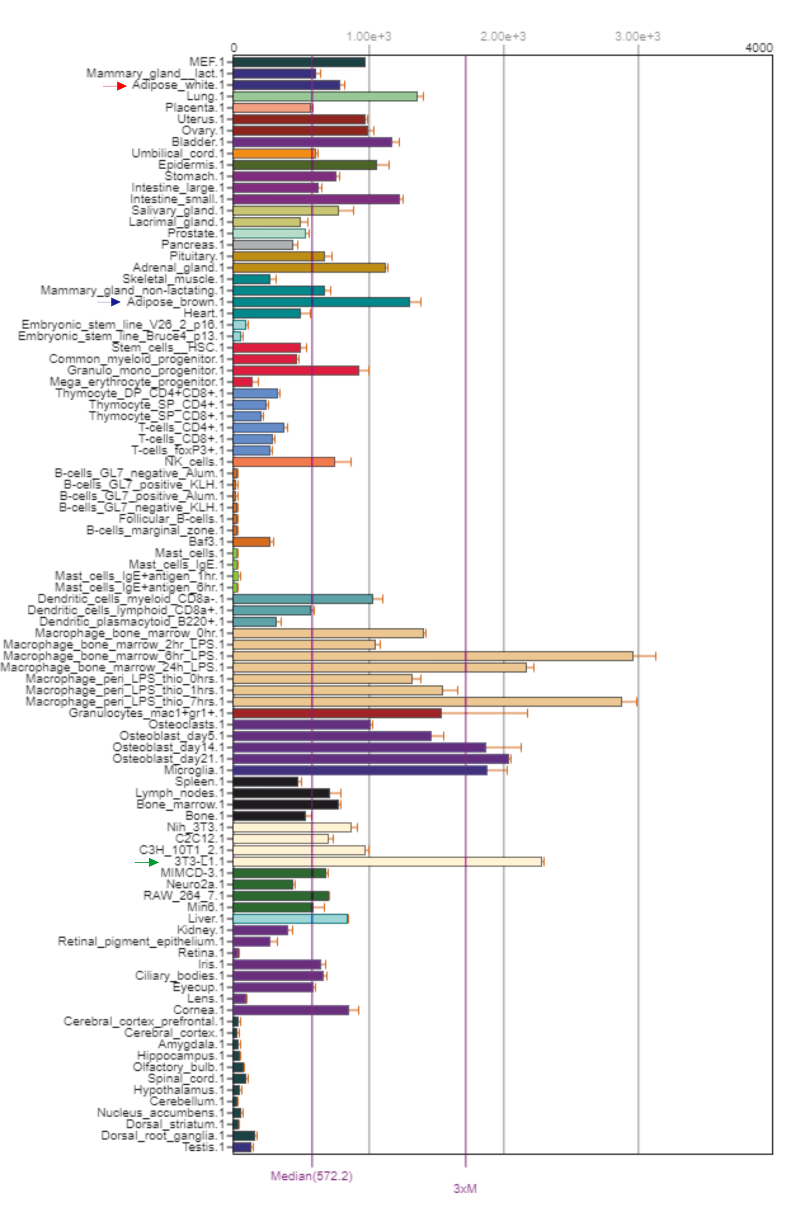


**Supplementary Fig. 2. Gene expression profile of Tnfsrf1a in mouse.**

The Online Biology Gene Portal System (BioGPS) revealed Tnfrsf1a highly expressed in white adipose tissue (red arrow), brown adipose tissue (blue arrow) and 3T3-L1 adipocytes (green arrow).

**
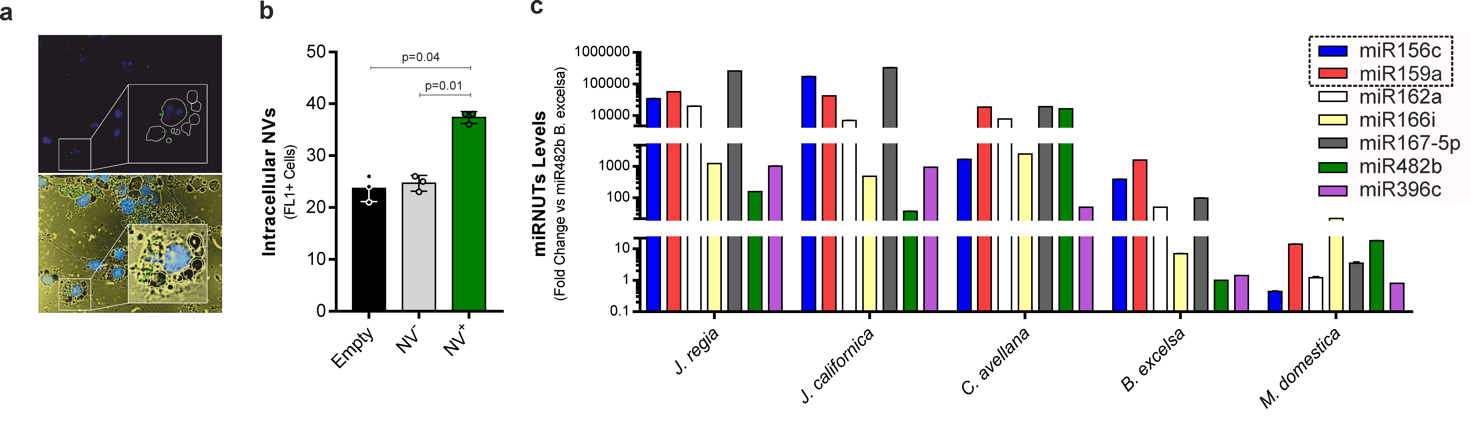
**

**Supplementary Fig. 3. NV Isolation, miRNA profiling and uptake in adipocytes.**

(**a, b**) NVs were stained with a specific probe against RNA and their adipocyte uptake was monitored by fluorescent microscopy (a) or cytofluorimetry (b).

**(c)** Determination of the presence of representative miR family members in nut NVs through RT-qPCR.

Data are expressed as means ± S.D. (n = 3).


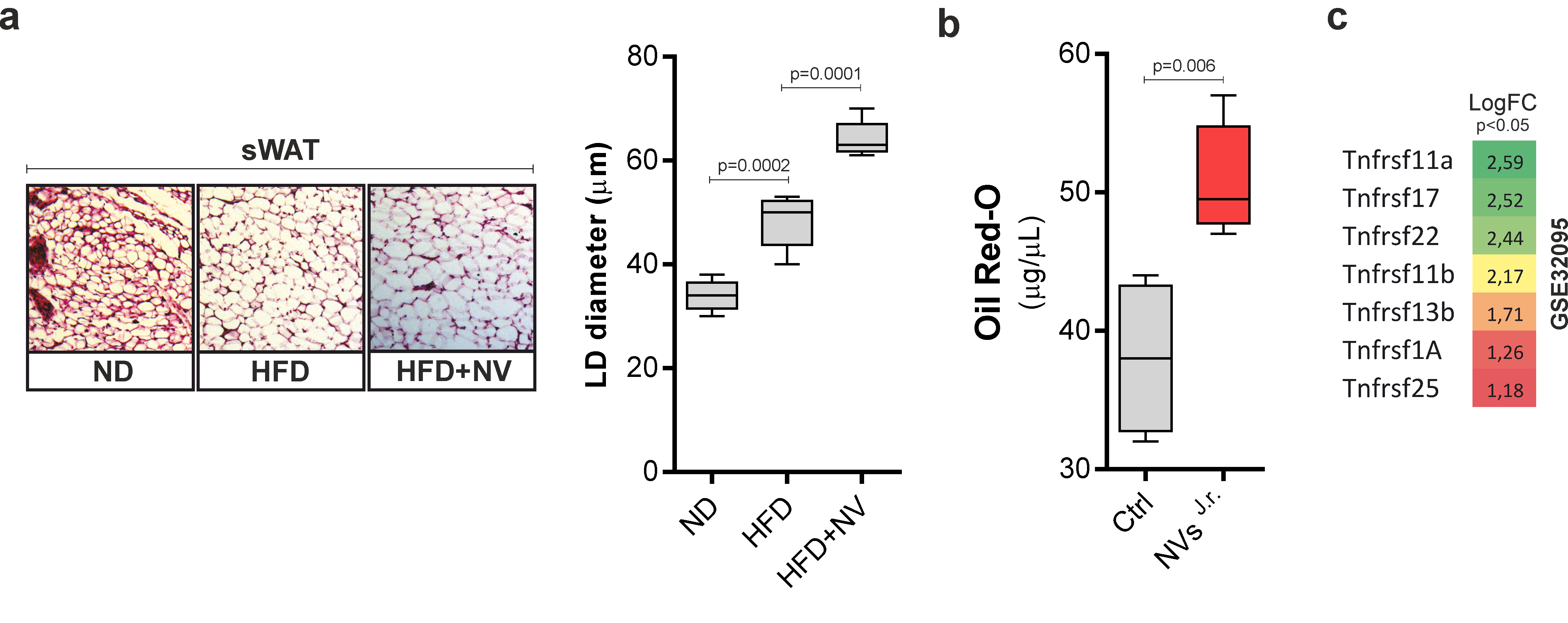


**Supplementary Fig. 4. Analysis of triglycerides amount, adipocytes size and Tnf receptors expression in cells or mice.**

(**a**) Representative images of H&E staining of subcutaneous adipose tissue (sWAT) from mice fed with normal diet (ND), high fat diet (HFD) or HFD supplemented with nut nanovesicles (HFD+NV). Measurement of LD diameter (right panel) was carried out in three fields for each sWAT histology using ImageJ (200 droplets total at least for each sample). Data are expressed as means ± S.D. (n = 3).

(**b**) Triglycerides amount was evaluated through Oil Red-O staining in T37i cells treated with NVs isolated from *J. regia* (NV^J.r.^). Data are expressed as means ± S.D. (n = 3)

(**c**) Gene expression levels of Tnf receptors, in visceral white adipose tissue of mice fed with high fat diet, was calculated by using a publicly available dataset (GSE32095). Data are expressed as log fold changes (LogFC) with respect to mice fed with normal diet (p<0.05).


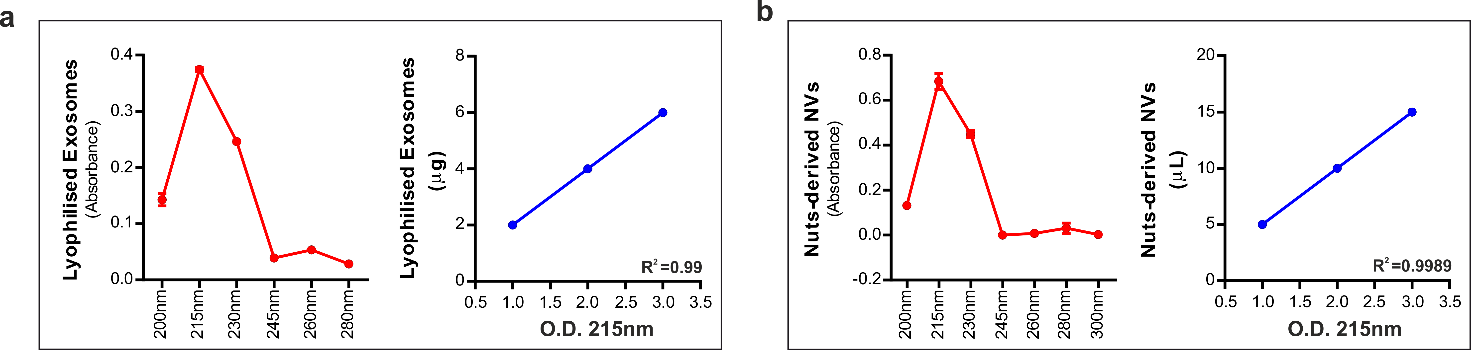


**Supplementary Fig. 5. Spectrophotometric quantitation of nuts NVs.**

(**a**) Absorption spectrum of purified human plasma exosomes (HansaBiomed) and reference curve construction at 215 nm (absorbance peak value).

(**b**) Absorption spectrum of purified nuts NVs and their quantification at 215 nm.

**Supplementary Fig. 6.** Uncropped gel images corresponding to main Figure 1.

**Supplementary Fig. 7.** Uncropped gel image corresponding to main Figure 4.

**
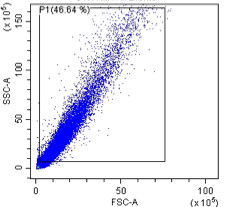
**

**Supplementary Fig. 8. FSC/SSC gating strategy relative to Fig. 4d.**

**Supplementary Fig. 9.** Uncropped gel images corresponding to main Figure 6

**Supplementary Table 1**. **GC-MS detected molecules**

|  | **Chemical name** | **PubChem CID** |
| --- | --- | --- |
| ***J. Regia*** | 2-phenylethyl hexanoate | 61384 |
|  | 2,4-DI-Tert-butylphenol | 7311 |
|  | Maltol | 8369 |
|  | p-Isopropylcinnamic acid | 719753 |
|  | Hexanoic Anhydride | 74918 |
|  | Isoamyl Decanoate | 75320 |
|  | Isoamyl Octanoate | 16255 |
|  | 3,7-Dimethyloctan-1-ol | 7792 |
|  | Valeric Acid | 7991 |
|  | 1,3-Ditert-butyl-5-methoxybenzene | 106440 |
|  | Palmitic Acid | 985 |
|  | Ethyl Palmitate | 12366 |
|  | Stearic Acid | 5281 |
|  | Ethyl Behenate | 22199 |
|  | Erucic Acid | 5281116 |
|  | 1-Tetracosanol | 10472 |
|  | Caproic Acid | 8892 |
|  | M-Tolualdehyde | 12105 |
|  | Precocene I | 28619 |
|  | P-Toluic acid | 7470 |
|  | 3,5-Di-tert-butyl-4-hydroxybenzyl acetate | 84404 |
| ***C. avellana*** | 3,7-Dimethyloctan-1-ol | 7792 |
|  | 3,5-Di-tert-butyl-4-hydroxybenzyl acetate | 84404 |
|  | Heneicosane | 12403 |
|  | Tridecanoic Acid | 12530 |
|  | Palmitic Acid | 985 |
|  | Nonadecanoic Acid | 12591 |
|  | Elaidic Acid | 637517 |
|  | Stearic Acid | 5281 |
|  | 1,3-Ditert-butyl-5-methoxybenzene | 106440 |
| ***J.californica*** | 1,3-Ditert-butyl-5-methoxybenzene | 106440 |
|  | 3,5-Di-tert-butyl-4-hydroxybenzyl acetate | 84404 |
|  | Palmitic Acid | 985 |
|  | Stearic Acid | 5281 |
|  | 3,7-Dimethyloctan-1-ol | 7792 |
|  | Butyric Anhydride | 7798 |
|  | Isovaleric Acid | 10430 |
|  | Isoamyl Isovalerate | 12613 |
|  | Ethyl Arachidate | 29009 |
|  | Hexanoic Anhydride | 74918 |
|  | Docosanol | 12620 |
|  | Ethyl Stearate | 8122 |

**Supplementary Table 2. miR sequences obtained from miRBase (**<http://www.mirbase.org>)

| **miRNA** | **Sequence** | **miRBase accession number** |
| --- | --- | --- |
| **156c** | 5’-UGACAGAAGAGAGUGAGCAC-3’ | MI0000180 |
| **159a** | 5’-UUUGGAUUGAAGGGAGCUCUA-3’ | MI0000189 |
| **162a** | 5’-UCGAUAAACCUCUGCAUCCAG-3’ | MI0000194 |
| **166i** | 5’-UCGGACCAGGCUUCAUUCCCC-3’ | MI0023025 |
| **167-5p** | 5’-UGAAGCUGCCAGCAUGAUCUU-3’ | MIMAT0016324 |
| **482b** | 5’-UCUUUCCUAUCCCUCCCAUUCC-3’ | MI0023126 |
| **396c** | 5’-UUCCACAGCUUUCUUGAACUU-3’ | MI0023095 |
