# Supplementary material for: Adipocyte metabolism is improved by TNF receptor-targeting small RNAs identified from dried nuts
Source: Commun Biol. 2019 Aug 21;2:317. doi: 10.1038/s42003-019-0563-7 (PMC6704100; doi:10.1038/s42003-019-0563-7)
Supplement: Supplementary file 2 — Reporting Summary [file 42003_2019_563_MOESM2_ESM.pdf]

nature research

Corresponding author(s): Katia Aquilano
Daniele Lettieri Barbato

Last updated by author(s): Jul 11, 2019

Reporting Summary

Nature Research wishes to improve the reproducibility of the work that we publish. This form provides structure for consistency and transparency in reporting. For further information on Nature Research policies, see Authors & Referees and the Editorial Policy Checklist.

Statistics

For all statistical analyses, confirm that the following items are present in the figure legend, table legend, main text, or Methods section.

n/a	Confirmed	
☐	☒	The exact sample size (*n*) for each experimental group/condition, given as a discrete number and unit of measurement
☐	☒	A statement on whether measurements were taken from distinct samples or whether the same sample was measured repeatedly
☐	☒	The statistical test(s) used AND whether they are one- or two-sided<br>*Only common tests should be described solely by name; describe more complex techniques in the Methods section.*
☒	☐	A description of all covariates tested
☐	☒	A description of any assumptions or corrections, such as tests of normality and adjustment for multiple comparisons
☐	☒	A full description of the statistical parameters including central tendency (e.g. means) or other basic estimates (e.g. regression coefficient) AND variation (e.g. standard deviation) or associated estimates of uncertainty (e.g. confidence intervals)
☐	☒	For null hypothesis testing, the test statistic (e.g. F, t, r) with confidence intervals, effect sizes, degrees of freedom and P value noted<br>*Give P values as exact values whenever suitable.*
☒	☐	For Bayesian analysis, information on the choice of priors and Markov chain Monte Carlo settings
☒	☐	For hierarchical and complex designs, identification of the appropriate level for tests and full reporting of outcomes
☒	☐	Estimates of effect sizes (e.g. Cohen's *d*, Pearson's *r*), indicating how they were calculated

Our web collection on statistics for biologists contains articles on many of the points above.

Software and code

Policy information about availability of computer code

Data collection	No software for data collection was used
Data analysis	Transcriptomics data (GSE32095) were analysed by FunRich 3.0; miRs-mRNA interactions were analysed by IntaRNA v2.0; CyKEGGParses (app of Cytoscape v3.6.1) was used to analyse the conservation rate of TNF-signaling pathway in adipocytes; miR-PREFeR pipeline was used to predict plant miRs from small-RNA seq. GraphPad Prism 6.0 was used to perform the statistical analyses.

For manuscripts utilizing custom algorithms or software that are central to the research but not yet described in published literature, software must be made available to editors/reviewers. We strongly encourage code deposition in a community repository (e.g. GitHub). See the Nature Research guidelines for submitting code & software for further information.

Data

Policy information about availability of data

All manuscripts must include a data availability statement. This statement should provide the following information, where applicable:

- Accession codes, unique identifiers, or web links for publicly available datasets
- A list of figures that have associated raw data
- A description of any restrictions on data availability

The datasets generated and analysed during the current study are publicly available on BioProject (ID PRJNA553332). All other data that support the findings of this study are available in the Supplemental materials or from the corresponding author upon reasonable request.

Field-specific reporting

Please select the one below that is the best fit for your research. If you are not sure, read the appropriate sections before making your selection.

☒ Life sciences ☐ Behavioural & social sciences ☐ Ecological, evolutionary & environmental sciences

For a reference copy of the document with all sections, see nature.com/documents/nr-reporting-summary-flat.pdf

Life sciences study design

All studies must disclose on these points even when the disclosure is negative.

Sample size	We have chosen a sample size of n=6 for each mouse group based on our previous published study (PMID: 29742122) in which this size was sufficient to find significant variations of fat mass and metabolic parameters (e.g. glycaemia and lipidemia) upon high fat diet.
Data exclusions	no data exclusion
Replication	All the experiments were replicated at least three times
Randomization	overall mice were randomly allocated
Blinding	All the experiments were blindly evaluated

Reporting for specific materials, systems and methods

We require information from authors about some types of materials, experimental systems and methods used in many studies. Here, indicate whether each material, system or method listed is relevant to your study. If you are not sure if a list item applies to your research, read the appropriate section before selecting a response.

Materials & experimental systems

n/a	Involved in the study	
☐	☒	Antibodies
☐	☒	Eukaryotic cell lines
☒	☐	Palaeontology
☐	☒	Animals and other organisms
☐	☒	Human research participants
☒	☐	Clinical data

Methods

n/a	Involved in the study	
☒	☐	ChIP-seq
☐	☒	Flow cytometry
☒	☐	MRI-based neuroimaging

Antibodies

Antibodies used	NFkBp65: sc-372 and sc-135769 (Santa Cruz Biotechnology); alpha-actinin: #3134 (Cell Signalling Technology); p-Akt1/2/3 (ser 473): sc-7985-R and sc-8312 (Santa Cruz Biotechnology); HSP60 (H-300): sc-13966 (Santa Cruz Biotechnology); Tubulin (H-300): sc-5546 (santa Cruz Biotechnology); Tom20 (FL-145): sc-11415 (Santa Cruz Biotechnology); Actin (H-196): sc-7210 (Santa Cruz Biotechnology); TNF receptor 1: Cat no. GTX33550 (GeneTex)
Validation	All antibodies were polyclonal antibodies with Rabbit as host. Moreover, all antibodies were validated for mouse reactivity by the manufacturer.

Eukaryotic cell lines

Policy information about cell lines

Cell line source(s)	3T3-L1 and RAW 264.7 were from ATCC; T37i were gently provided by Prof. Marc Lombes (INSERM, Paris, France)
Authentication	none of the cell lines used were authenticated
Mycoplasma contamination	All the used cell lines were negative for mycoplasma contamination (assessed by PCR or DAPI staining)
Commonly misidentified lines (See ICLAC register)	none

Animals and other organisms

Policy information about studies involving animals; ARRIVE guidelines recommended for reporting animal research

Laboratory animals	Female C67BL/6J 2-months age
Wild animals	none
Field-collected samples	Mice were maintained at 25°C with 12 hours light/dark cycles and had free access to food and water.
Ethics oversight	Animal experimentation was approved by The University Animal Welfare Committe - OPBA, Tor Vergata University; and Italian Ministry of Health (authorization n°378/2017-pr).

Note that full information on the approval of the study protocol must also be provided in the manuscript.

Human research participants

Policy information about studies involving human research participants

Population characteristics	We isolated macrophages from buffy coats of adult and anonymous human healthy individuals
Recruitment	Buffy coats of healthy donors, who gave their written informed consent to donate the non-clinically usable components of their blood for scientific research, were obtained from the Blood Transfusion Unit of Policlinico “Umberto I” in Rome.
Ethics oversight	The present study, which is based on non-clinical in vitro research, did not require any specific approval from an Ethical Committee, according to the current italian law (decree by Ministero della Salute by February 8th, 2013, published on Gazzetta Ufficiale della Repubblica Italiana no. 96 of April 24th, 2013, and legislative decree no. 211 of June 24th, 2003, published on Gazzetta Ufficiale della Repubblica Italiana no. 184 of August 9th, 2003).

Note that full information on the approval of the study protocol must also be provided in the manuscript.

Flow Cytometry

Plots

Confirm that:

☒ The axis labels state the marker and fluorochrome used (e.g. CD4-FITC).

☒ The axis scales are clearly visible. Include numbers along axes only for bottom left plot of group (a 'group' is an analysis of identical markers).

☒ All plots are contour plots with outliers or pseudocolor plots.

☒ A numerical value for number of cells or percentage (with statistics) is provided.

Methodology

Sample preparation	Mouse 3T3-L1 and T37i cell lines were probed with a glucose fluorescent analogue (2-NBDG) or with a TNF-alpha antibody (BD Bioscence; Rat monoclonal, clone MP6-XT22, #554419)
Instrument	CytoFlex (Beckman Coulter)
Software	Cytexpert 1.2 (Beckman Coulter)
Cell population abundance	Only cell lines were analysed
Gating strategy	The preliminary FSC/SSC gates were builded on a homogeneous cell population in order to eliminate cell debris. The boundaries between TNF-positive and negative cells were defined based on cells without TNF antibody staining (blank). The same gating strategy was performed for experiments with 2-NBDG.

☒ Tick this box to confirm that a figure exemplifying the gating strategy is provided in the Supplementary Information.
